# Supplementary material for: Structural basis for human sterol isomerase in cholesterol biosynthesis and multidrug recognition
Source: Nat Commun. 2019 Jun 5;10:2452. doi: 10.1038/s41467-019-10279-w (PMC6549186; doi:10.1038/s41467-019-10279-w)
Supplement: Supplementary file 1 — Supplementary Information [file 41467_2019_10279_MOESM1_ESM.pdf]

Structural basis for human sterol isomerase in cholesterol biosynthesis and multidrug recognition

Tao Long, Abdirahman Hassan, Bonne M Thompson, Jeffrey G McDonald, Jiawei Wang and Xiaochun Li

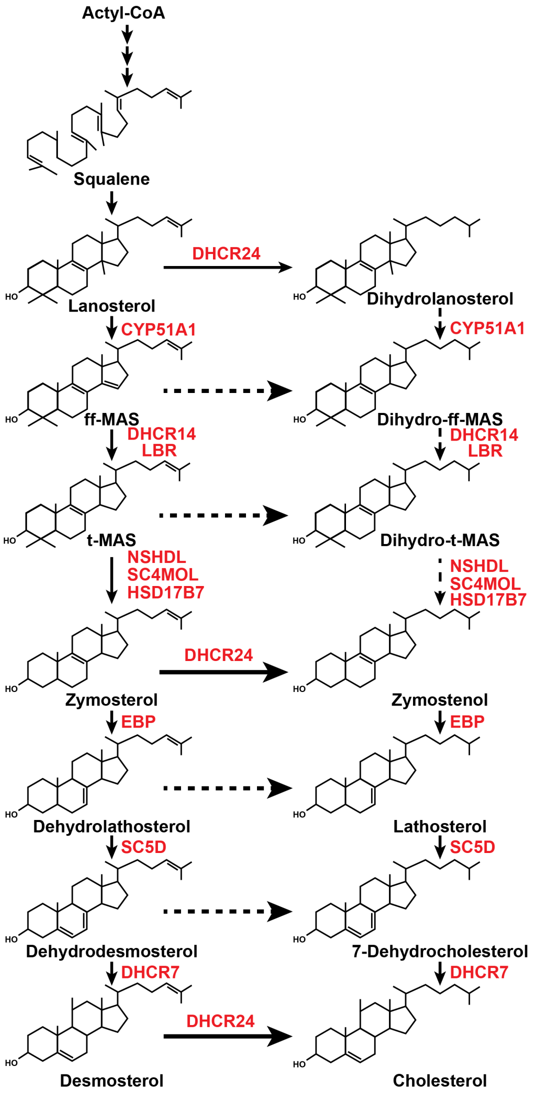

Supplementary Figure 1 Cholesterol biosynthesis pathway and membrane enzymes. Acetyl-CoA is the precursor for cholesterol biosynthesis. After several reactions, the intermediate lanosterol is synthesized. Conversion of lanosterol to cholesterol involves many reactions catalyzed by membrane enzymes (red). The left lane denotes the Bloch pathway; the right lane denotes the Kandutsch-Russell pathway. 24,25-double bond desaturation can occur at any step between lanosterol and desmosterol in this pathway, but in most tissues desaturation does not occur until after demethylation is complete (after t-MAS) [1].

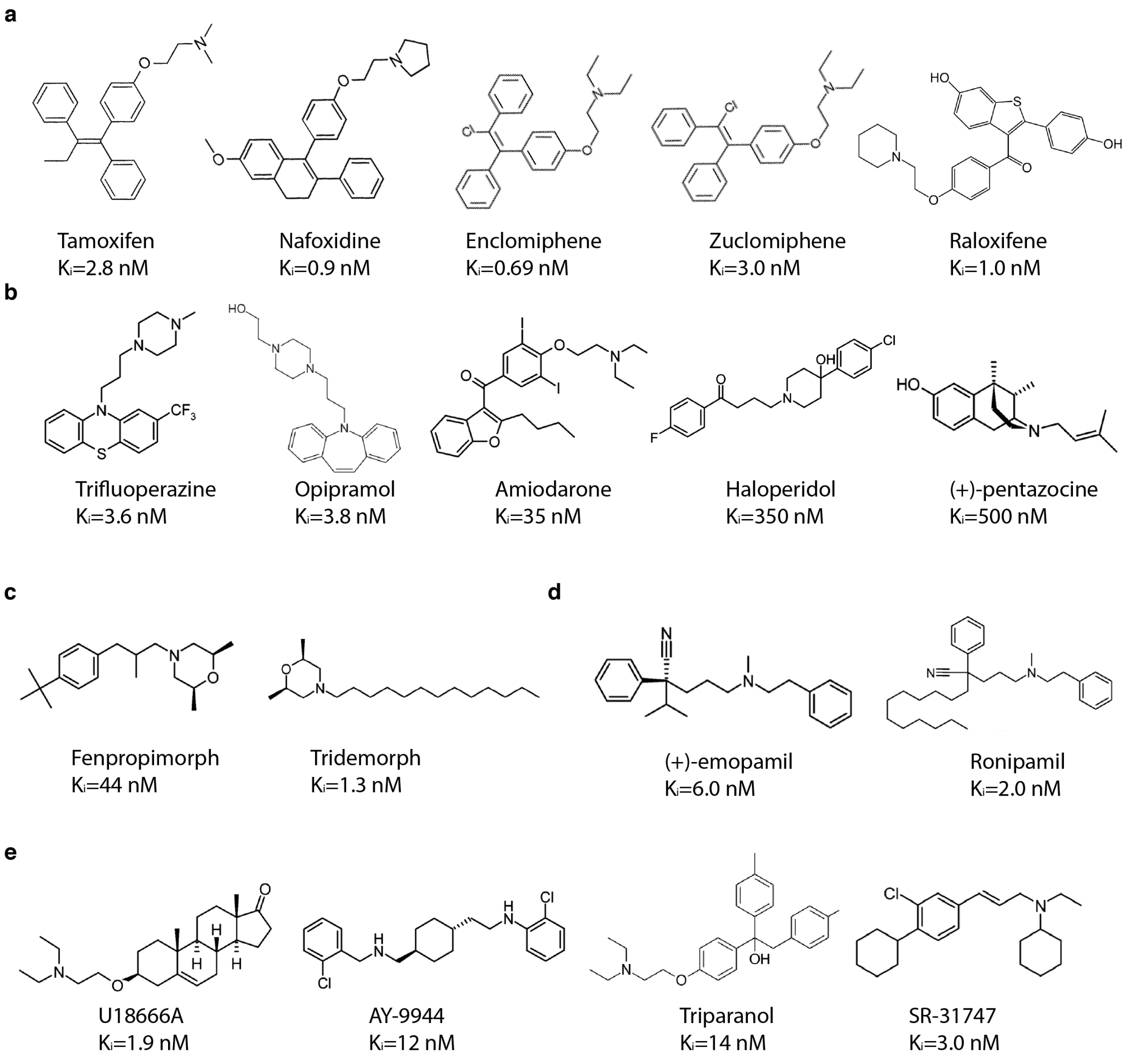

Supplementary Figure 2 Summary of pharmacologically active compounds that bind to human EBP protein.

a, Anti-tumor drugs. **b,** Neuroprotective drugs. **c,** Fungicides. **d,** Calcium channel blockers. **e,** Sterol biosynthesis inhibitors. K_i values are from the previous reports [2,3].

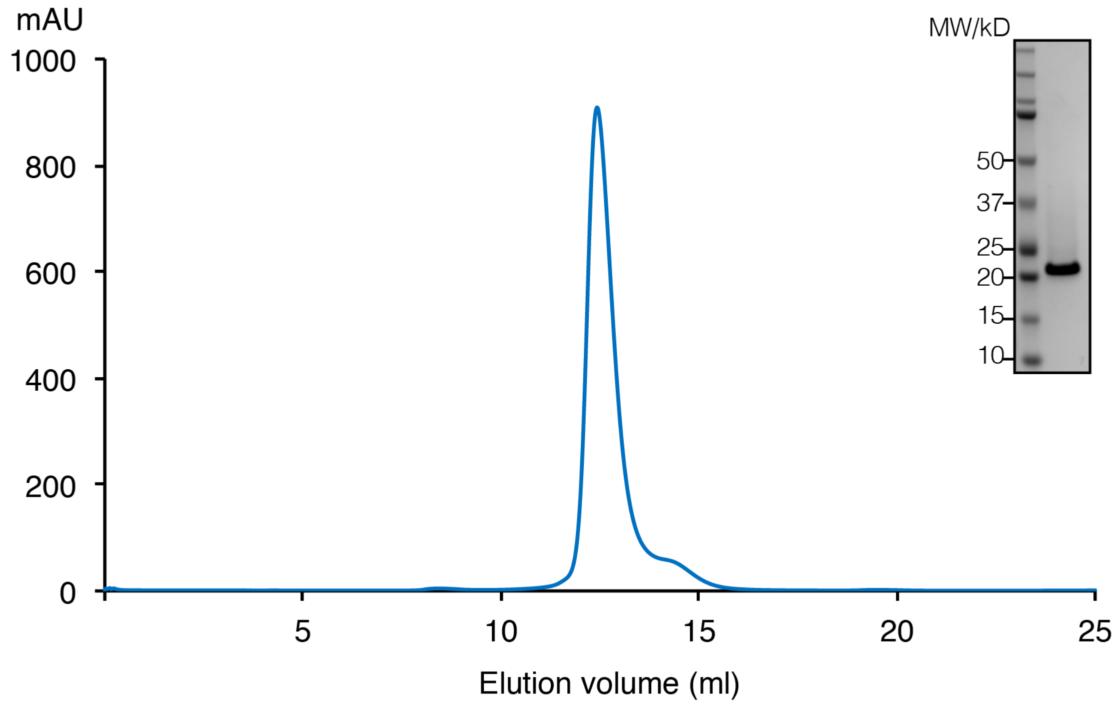

Supplementary Figure 3 Expression and purification of human EBP protein.

Representative Superdex 200 increase 10/30 gel-filtration chromatogram of human EBP.

Peak fraction of human EBP is shown on SDS-PAGE with molecular markers.

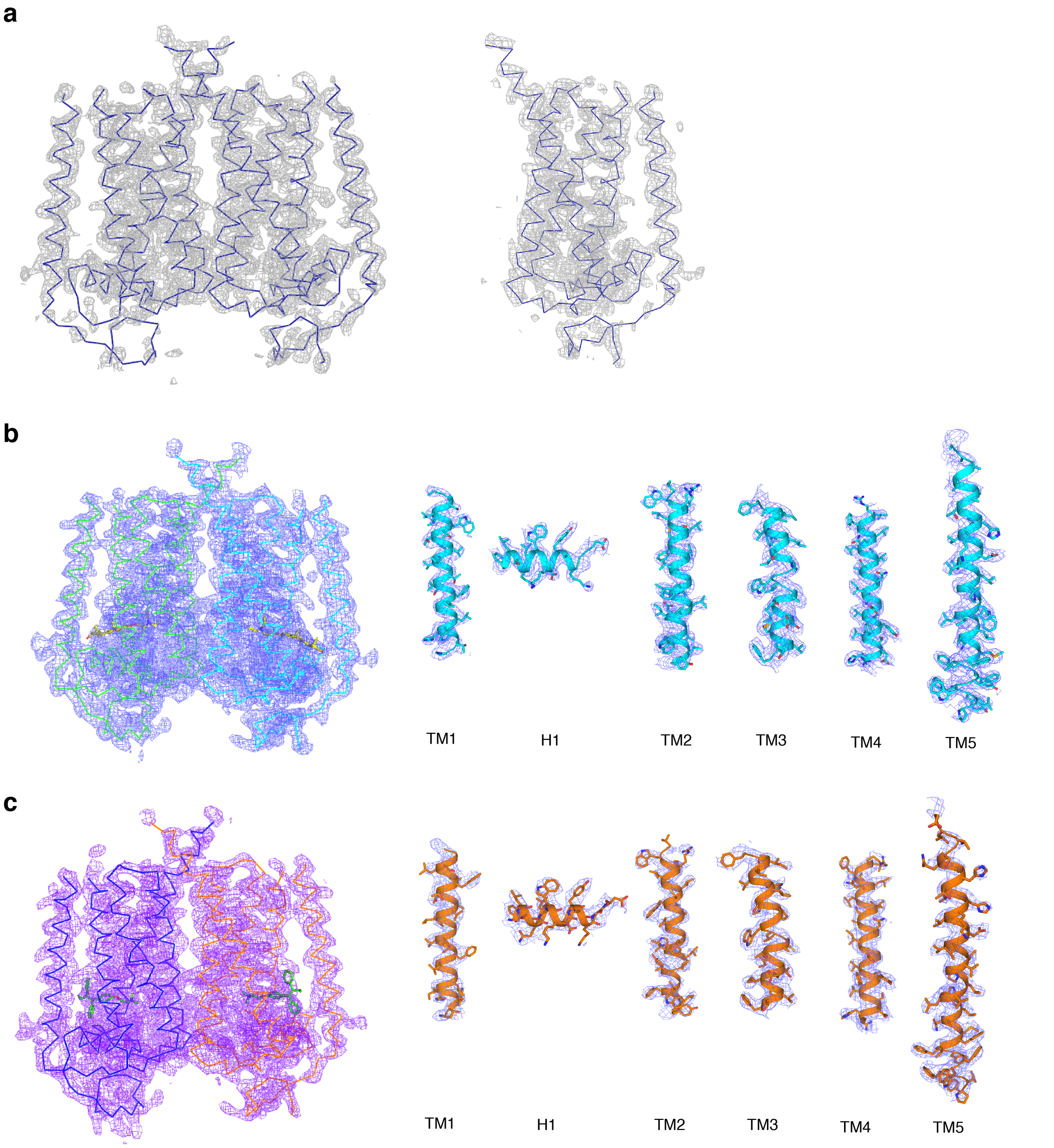

Supplementary Figure 4 The *2Fo-Fc* electron density of EBP.

a, An overall view of the experimental electron density, contoured at 1.5 σ, in one unit cell. **b,** The *2Fo-Fc* electron density map of EBP with U18666A, contoured at 1.0 σ. An overall view of the EBP dimer is shown (left). A representative view of the helices of Molecule A is shown (right). **c,** The *2Fo-Fc* electron density map of EBP with tamoxifen, contoured at 1.0 σ. An overall view of the EBP dimer is shown (left). A representative view of the helices of Molecule A is shown (right).

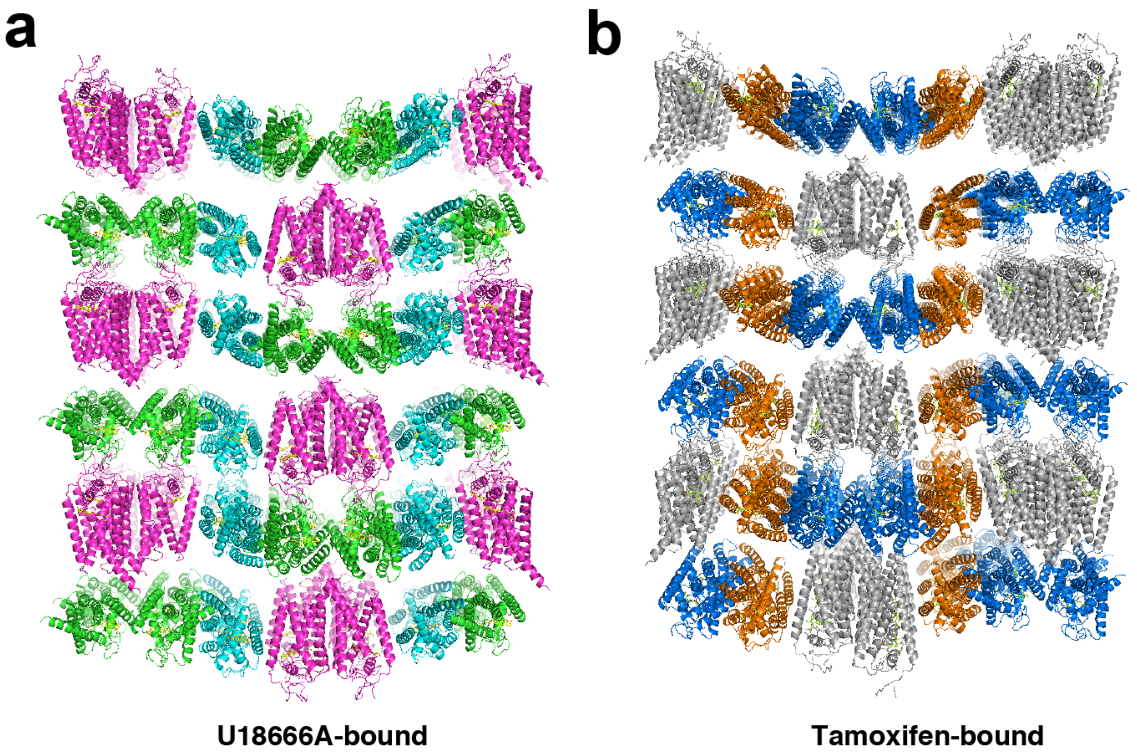

Supplementary Figure 5 Crystal packing of human EBP protein.

a, EBP with U18666A. **b,** EBP with tamoxifen. The three molecules of EBP protein in an asymmetric unit are indicated in the different colors.

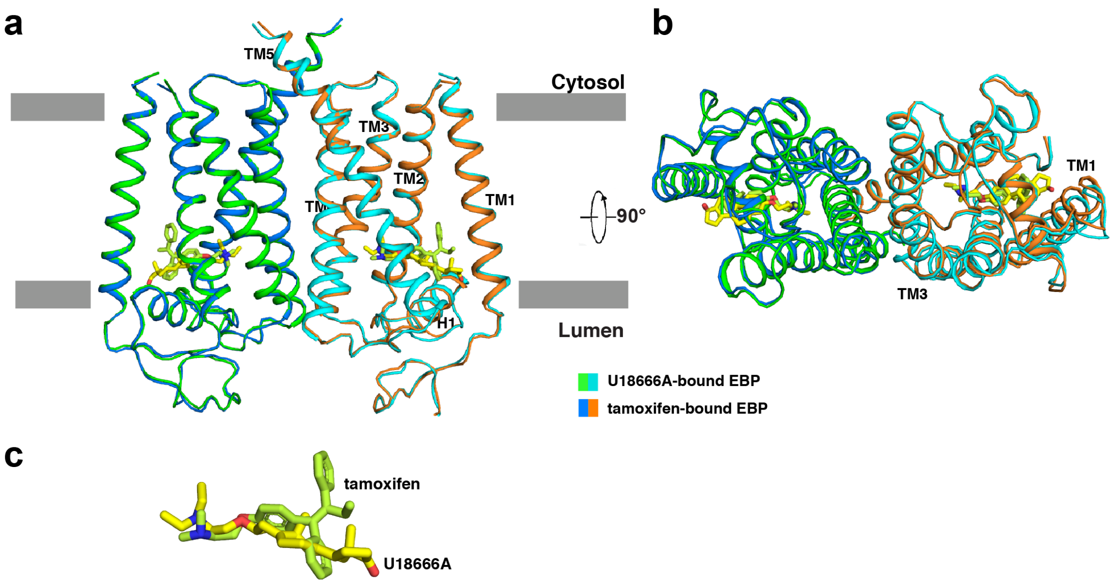

Supplementary Figure 6 Structural comparison of U18666A-bound and tamoxifen-bound EBP.

a, Structural comparison from parallel to the membrane view. **b,** Structural comparison from lumen view. **c,** The comparison of U18666A and tamoxifen in the structures.

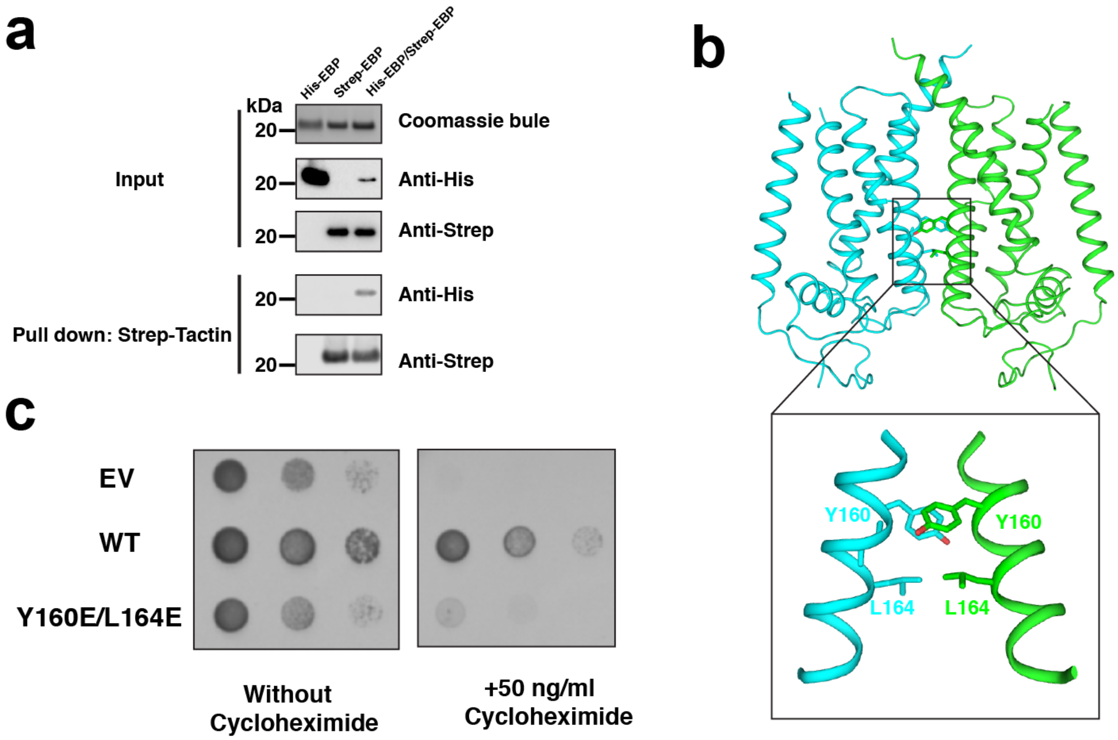

Supplementary Figure 7 Physiological function of EBP dimer

a, The EBP dimer is verified by pull down assay. Protein with both His and Strep tag was expressed by cells which were co-infected with two baculoviruses (see Methods) and purified by Strep-Tactin affinity column. **b,** The dimer interface of EBP. The key residues that form the interface are shown as sticks**. c,** Yeast complementation assay. EBP with dimerization mutations can rescue the growth of ΔErg2 *Saccharomyces cerevisiae*, but these mutants are not as efficient as WT. Growth of yeast expressing EBP mutant in the presence of 50 ng/ml concentrations of cycloheximide for 24 to 48 hours. Source data are provided as a Source Data file.

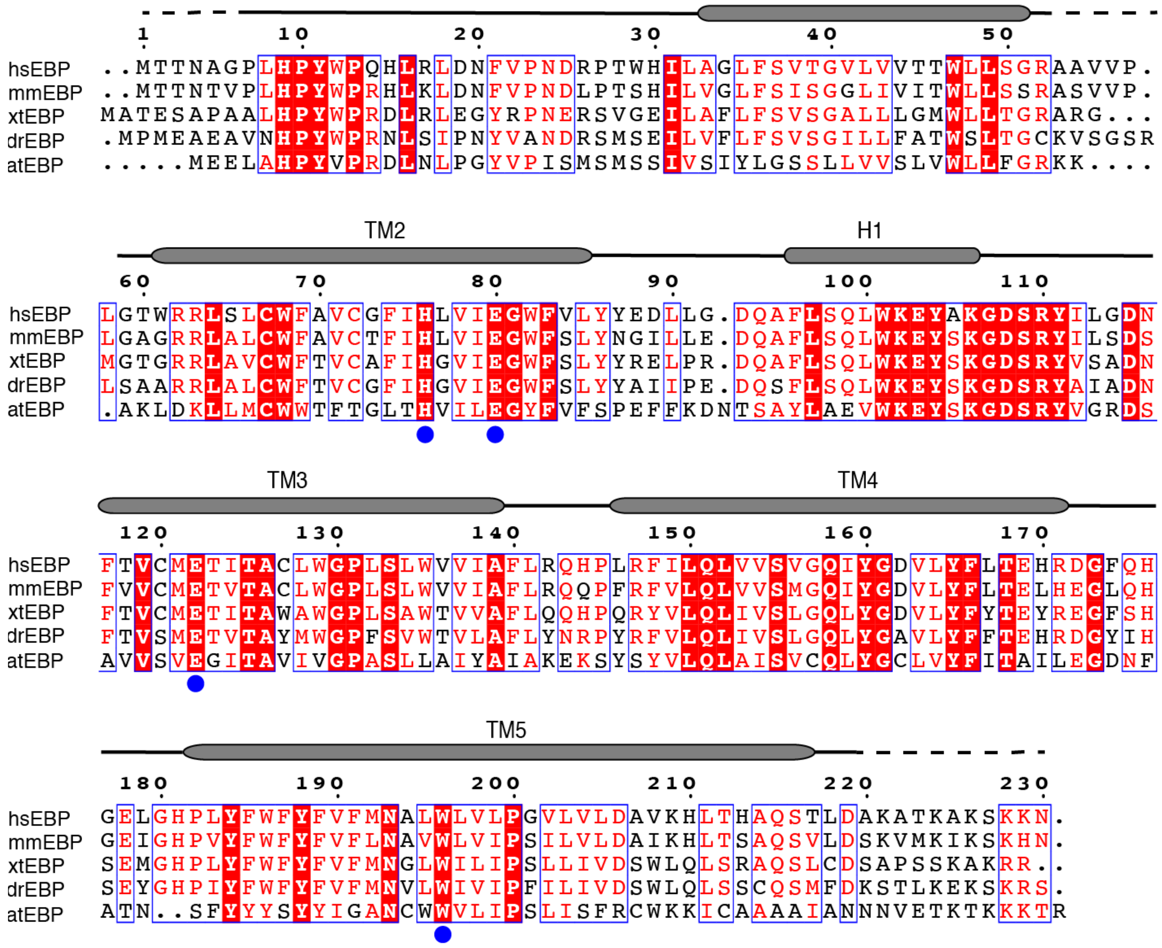

Supplementary Figure 8 Sequence alignment of human EBP protein with different species EBP proteins. The residue numbers of hsEBP are indicated above the protein sequence. The secondary structures are labeled. Residues under the dashed lines are excluded from the 3D reconstruction. The catalytic residues for the reaction are indicated by blue balls. Species label: hs-*Homo sapiens*; mm-*Mus musculus*; xt-*Xenopus tropicalis*; dr-*Danio rerio*; at-*Arabidopsis thaliana.*

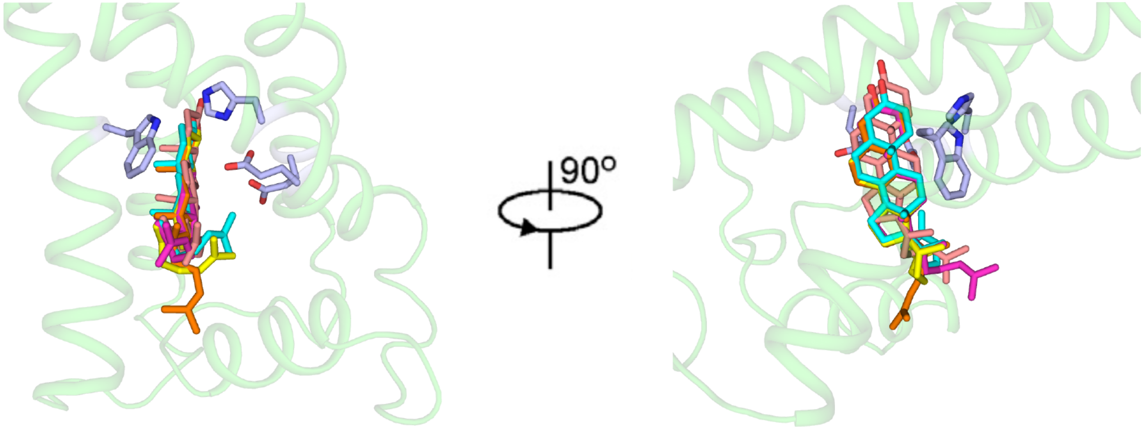

Supplementary Figure 9 Molecular Docking Simulations for binding of Δ^{8}-sterol to EBP.

The top 5 scoring poses of Δ^{8}-sterol bound to EBP are overlaid with key residues represented as sticks (light blue). Color: pose 1, orange; pose 2, cyan; pose 3, magenta; pose 4, yellow; pose 5, wheat.

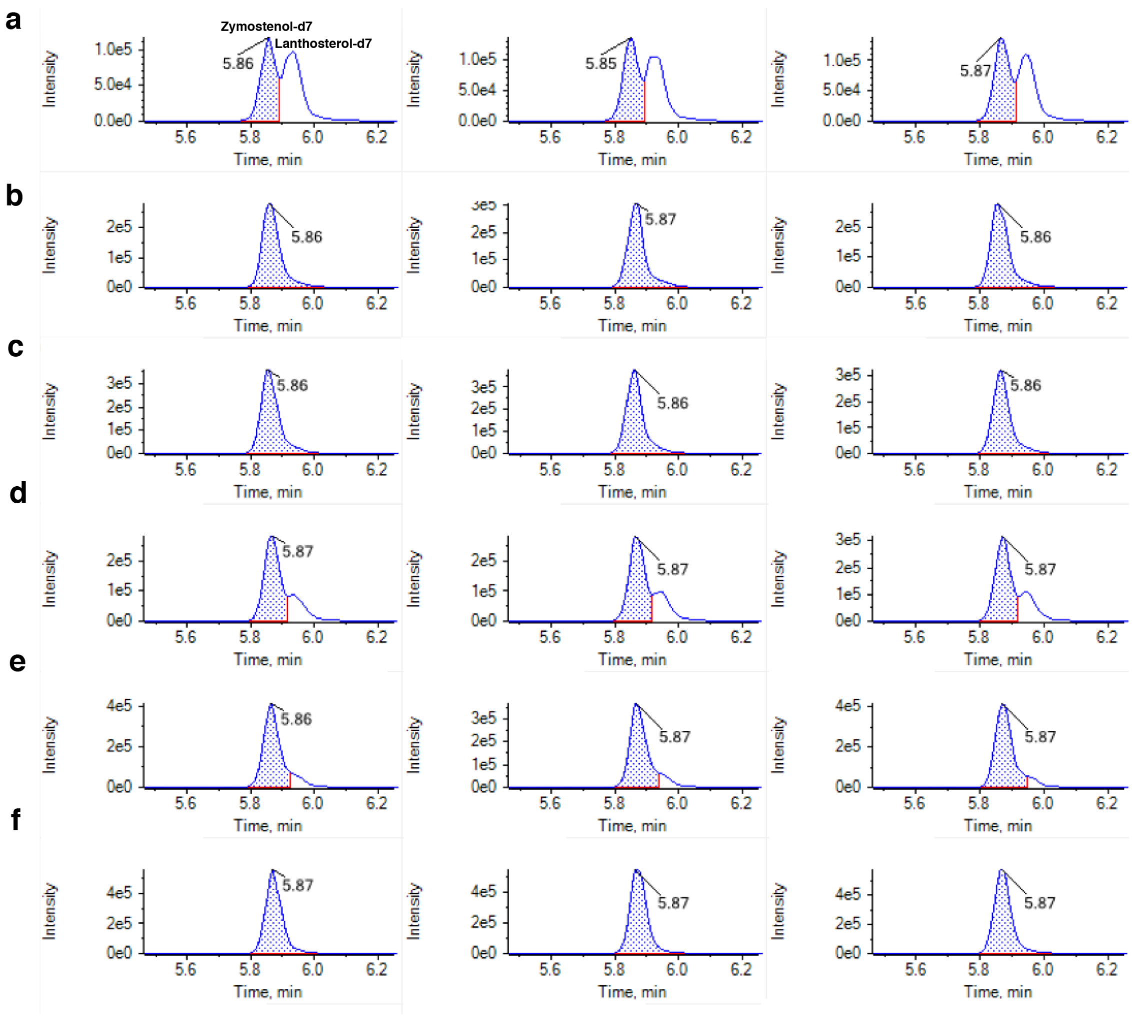

Supplementary Figure 10 LC-MS/MS chromatography of EBP activity assays. **a,** wild type. **b,** mutant with H76A. **c,** mutant with E80A. **d,** mutant with E122A. **e,** mutant with W196A. **f,** No protein. The peak of the precursor, deuterium labeled zymostenol, is colored in blue; the peak of the product, lanthosterol, is colored in white. LC-MS/MS results with three independent repeats.

Supplementary Table 1. Data collection, phasing and refinement statistics

	U18666A-bound (Se-labeled)	Tamoxifen-bound (Native form)	Se-SAD
**Data collection**			
Space group	$P2_12_12$	$P2_12_12$	$P2_12_12$
Cell dimensions			
a, *b*, *c* (Å)	194.566, 67.823, 96.858	192.837, 66.829, 98.678	194.338, 67.923, 99.731
α, β, γ (°)	90, 90, 90	90, 90, 90	90, 90, 90
			Peak
Wavelength	0.97927	0.97927	0.97910
Resolution (Å)	50~3.15 (3.26~3.15)	50~3.45 (3.51~3.45)	50~3.50 (3.63~3.50)
R_{merge}(%)	8.6 (>1)	11.7 (>1)	13.2 (>1)
$I / \sigma I$	30.0 (1.2)	17.3 (1.0)	21.1 (1.3)
Completeness (%)	99.8 (99.6)	99.6 (99.7)	99.6 (99.8)
Redundancy	12.9 (11.9)	6.3 (5.7)	43.5 (30.1)
**Refinement**			
Resolution (Å)	50~3.20	50~3.50	
No. reflections	28389	13685	
R_{work} / R_{free}	26.85 / 31.91	27.25 / 32.59	
No. atoms	5208	5208	
Protein	5124	5124	
Ligand/ion	84	84	
Water	0	0	
B-factors			
Protein	56.30	35.37	
Ligand/ion	61.35	36.32	
Water	N/A	N/A	
R.m.s deviations			
Bond lengths (Å)	0.004	0.004	
Bond angles (°)	0.927	0.927	

Values in parentheses are for the highest resolution shell. R_{free} was calculated with 5% of the reflections selected in the thin shell.

Supplementary Table 2. Disease-related mutations and their probably effect and consequence.

Mutation	Probably effect	Likely consequence
L18P[4]	Block the solvent entry	Non-functional protein
W47C[5]	To be determined	Unknown
W47R[6]	To be determined	Unknown
R62W[7]	To be determined	Unknown
L66P[8]	To be determined	Unknown
C72R[9]	Sterol binding /entry	Non-functional protein
C72Y[7]	Sterol binding /entry	Non-functional protein
G73E[9]	Sterol binding /entry	Non-functional protein
I75N[10,11]	Sterol binding /entry	Non-functional protein
E80K[12,13]	Catalytic activity	Non-functional protein
W82C[14,15]	Sterol binding /entry	Non-functional protein
L100P[9]	Sterol binding /entry	Non-functional protein
W101C[9]	Sterol binding /entry	Non-functional protein
E103K[16]	Block the solvent entry	Non-functional protein
Y104H[9]	Sterol binding /entry	Non-functional protein
Y104C[9]	Sterol binding /entry	Non-functional protein
A105D[9]	Block the solvent entry	Non-functional protein
G107E[9]	Block the solvent entry	Non-functional protein
R110Q[16,17]	Block the solvent entry	Non-functional protein
Y111H[9]	Sterol binding /entry	Non-functional protein
G130V[7]	Sterol binding /entry	Non-functional protein
S133R[8,13]	To be determined	Unknown
F140C[9]	To be determined	Unknown
R147C[9]	To be determined	Unknown
R147H[14-16]	To be determined	Unknown
G157S[7]	Dimerization	Destabilize the protein or cause folding defect
G161R[9]	Dimerization	Destabilize the protein or cause folding defect
D162H[8]	Sterol binding /entry	Non-functional protein
L164P[16]	Dimerization	Destabilize the protein or cause folding defect
Y165C[14]	Sterol binding /entry	Non-functional protein
R171C[9]	Dimerization	Destabilize the protein or cause folding defect
R171H[9]	Dimerization	Destabilize the protein or cause folding defect
G173R[7]	To be determined	Unknown
H176R[9]	To be determined	Unknown
W196S[7]	Sterol binding /entry	Non-functional protein
L203P[15]	To be determined	Unknown

L211R[9]	Dimerization	Destabilize the protein or cause folding defect
T217M[9]	Dimerization	Destabilize the protein or cause folding defect

Supplementary Table 3. Lists of primers used in this study.

**Primer name**	**Sequences (5' – 3')**
EBP_Strep_Forward	AATGAATTCATGTGGTCACATCCGCAGTTCGAGAAAA CTACCAACGCGGGCCCC
EBP_Flag_Forward	CGCGGATCC GACTACAAAGACGATGACGACAAGACTA CCAACGCGGGCCCC
EBP_His_Forward	AATGAATTCATGCACCACCACCACCACCACCACCACAC TACCAACGCGGGCCCC
EBP_Reverse	AATGCGGCCGCTCAGTTCTTCTTGCTCTTGGC
EBP_H76A_Forward	GTGTGTGGGTTCATTGCCCTGGTGATCGAGGGC
EBP_H76A_Reverse	GCCCTCGATCACCAGGGCAATGAACCCACACAC
EBP_E80A_Forward	ATTCACCTGGTGATCGCGGGCTGGTTCGTTCTC
EBP_E80A_Reverse	GAGAACGAACCAGCCCGCGATCACCAGGTGAAT
EBP_E122A_Forward	TTCACAGTGTGCATGGCAACCATCACAGCTTGC
EBP_E122A_Reverse	GCAAGCTGTGATGGTTGCCATGCACACTGTGAA
EBP_N193A_Forward	TACTTTGTCTTCATGGCTGCCCTGTGGCTGGTG
EBP_N193A_Reverse	CACCAGCCACAGGGCAGCCATGAAGACAAAGTA
EBP_W196A_Forward	TTCATGAATGCCCTGGCGCTGGTGCTGCCTGGA
EBP_W196A_Reverse	TCCAGGCAGCACCAGCGCCAGGGCATTCATGAA
EBP_Y160E/L164E_Forward	TCTGTGGGCCAGATCGAGGGGGATGTGGAGTACTTCCT GACAGAG
EBP_Y160E/L164E_Reverse	CTCTGTCAGGAAGTACTCCACATCCCCCTCGATCTGGCC CACAGA

References:

1 Mitsche, M. A., McDonald, J. G., Hobbs, H. H. & Cohen, J. C. Flux analysis of cholesterol biosynthesis in vivo reveals multiple tissue and cell-type specific pathways. *eLife* **4**, e07999, doi:10.7554/eLife.07999 (2015).

2 Laggner, C. *et al.* Discovery of high-affinity ligands of sigma1 receptor, ERG2, and emopamil binding protein by pharmacophore modeling and virtual screening. *Journal of medicinal chemistry* **48**, 4754-4764, doi:10.1021/jm049073+ (2005).

3 Moebius, F. F. *et al.* Pharmacological analysis of sterol delta8-delta7 isomerase proteins with [3H]ifenprodil. *Molecular pharmacology* **54**, 591-598 (1998).

4 Milunsky, J. M., Maher, T. A. & Metzenberg, A. B. Molecular, biochemical, and phenotypic analysis of a hemizygous male with a severe atypical phenotype for X-linked dominant Conradi-Hunermann-Happle syndrome and a mutation in EBP. *American journal of medical genetics. Part A* **116a**, 249-254, doi:10.1002/ajmg.a.10849 (2003).

5 Furtado, L. V. *et al.* A novel X-linked multiple congenital anomaly syndrome associated with an EBP mutation. *American journal of medical genetics. Part A* **152a**, 2838-2844, doi:10.1002/ajmg.a.33674 (2010).

6 Hartill, V. L. *et al.* An unusual phenotype of X-linked developmental delay and extreme behavioral difficulties associated with a mutation in the EBP gene. *American journal of medical genetics. Part A* **164a**, 907-914, doi:10.1002/ajmg.a.36368 (2014).

7 Herman, G. E. *et al.* Characterization of mutations in 22 females with X-linked dominant chondrodysplasia punctata (Happle syndrome). *Genetics in medicine : official journal of the American College of Medical Genetics* **4**, 434-438, doi:10.109700125817-200211000-00006 (2002).

8 Whittock, N. V. *et al.* Novel mutations in X-linked dominant chondrodysplasia punctata (CDPX2). *The Journal of investigative dermatology* **121**, 939-942, doi:10.1046/j.1523-1747.2003.12489.x (2003).

9 Landrum, M. J. *et al.* ClinVar: improving access to variant interpretations and supporting evidence. *Nucleic acids research* **46**, D1062-d1067, doi:10.1093/nar/gkx1153 (2018).

10 Barboza-Cerda, M. C., Wong, L. J., Martinez-de-Villarreal, L. E., Zhang, V. W. & Dector, M. A. A novel EBP c.224T>A mutation supports the existence of a male-specific disorder independent of CDPX2. *American journal of medical genetics. Part A* **164a**, 1642-1647, doi:10.1002/ajmg.a.36508 (2014).

11 Barboza-Cerda, M. C., Campos-Acevedo, L. D., Rangel, R., Martinez-de-Villarreal, L. E. & Dector, M. A. A novel phenotype characterized by digital abnormalities, intellectual disability, and short stature in a Mexican family maps to Xp11.4-p11.21. *American journal of medical genetics. Part A* **161a**, 237-243, doi:10.1002/ajmg.a.35743 (2013).

12 Aughton, D. J., Kelley, R. I., Metzenberg, A., Pureza, V. & Pauli, R. M. X-linked dominant chondrodysplasia punctata (CDPX2) caused by single gene mosaicism in a male. *American journal of medical genetics. Part A* **116a**, 255-260, doi:10.1002/ajmg.a.10852 (2003).

13 Braverman, N. *et al.* Mutations in the gene encoding 3 beta-hydroxysteroid-delta 8, delta 7-isomerase cause X-linked dominant Conradi-Hunermann syndrome. *Nature genetics* **22**, 291-294, doi:10.1038/10357 (1999).

14 Shirahama, S. *et al.* Skewed X-chromosome inactivation causes intra-familial phenotypic variation of an EBP mutation in a family with X-linked dominant chondrodysplasia punctata. *Human genetics* **112**, 78-83, doi:10.1007/s00439-002-0844-x (2003).

15 Has, C. *et al.* Gas chromatography-mass spectrometry and molecular genetic studies in families with the Conradi-Hunermann-Happle syndrome. *The Journal of investigative dermatology* **118**, 851-858, doi:10.1046/j.1523-1747.2002.01761.x (2002).

16 Canueto, J. *et al.* Clinical, molecular and biochemical characterization of nine Spanish families with Conradi-Hunermann-Happle syndrome: new insights into X-linked dominant chondrodysplasia punctata with a comprehensive review of the literature. *The British journal of dermatology* **166**, 830-838, doi:10.1111/j.1365-2133.2011.10756.x (2012).

17 Derry, J. M. *et al.* Mutations in a delta 8-delta 7 sterol isomerase in the tattered mouse and X-linked dominant chondrodysplasia punctata. jderry@immunex.com. *Nature genetics* **22**, 286-290, doi:10.1038/10350 (1999).
